# Effects of Surface Treatments on Innovative Additively Manufactured Scaffolds: Implications for Biocompatibility in Bone Tissue Engineering

**DOI:** 10.3390/life15111755

**Published:** 2025-11-15

**Authors:** Qun Zhao, Florian Fischer, Maximilian Voshage, Lucas Jauer, Alexander Kopp, Maximilian Praster, Rald Victor Maria Groven, Johannes Henrich Schleifenbaum, Jörg Eschweiler, Philipp Kobbe, Eva Miriam Buhl, Frank Hildebrand, Elizabeth R. Balmayor, Johannes Greven

**Affiliations:** 1 Experimental Orthopaedics and Trauma Surgery, University Hospital RWTH Aachen, 52074 Aachen, Germany; 2 Chair for Digital Additive Production DAP, RWTH Aachen University, 52074 Aachen, Germany; 3 Meotec GmbH, 52068 Aachen, Germany; 4 Department of Orthopaedics, Trauma and Reconstructive Surgery, University Hospital RWTH Aachen, 52074 Aachen, Germany; 5 Department of Trauma and Reconstructive Surgery, BG Hospital Bergmannstrost Halle, 06112 Halle (Saale), Germany; 6 Department of Trauma and Reconstructive Surgery, University Hospital of the Martin Luther University Halle, 06120 Halle (Saale), Germany; 7 Electron Microscopy Facility, Institute of Pathology, University Hospital RWTH Aachen, 52074 Aachen, Germany; 8 Department of Thoracic Surgery, University Hospital RWTH Aachen, 52074 Aachen, Germany

**Keywords:** additively manufactured, biocompatibility, bone tissue engineering

## Abstract

**Purpose**: The increasing demand for alternatives to autologous and resorbable bone grafts in the treatment of bone defects is driving research efforts. This study aims to evaluate the effects of different surface treatments on zinc-1%-magnesium (Zn-1Mg) alloy scaffolds on chondrocytes and osteoblasts, focusing on cytotoxicity, biocompatibility, and cell proliferation. **Methods**: Zn-1Mg alloy disks were manufactured additively by the powder bed fusion of metals using a laser beam (PBF-LB/M) and underwent different distinct surface treatments, including as-built treatment, sandblasting, Zn-1Mg-blasting, and electropolishing, respectively. Chondrocytes and osteoblasts were cultured separately on these additively manufactured Zn-1Mg alloy disks for 3, 7, and 14 days to assess biocompatibility and cellular growth. Cell viability, cytotoxicity, and proliferation were analyzed using DAPI staining, live/dead staining, fluorescence microscopy, and flow cytometry. Additionally, cellular morphology was investigated using Phalloidin/DAPI staining and scanning electron microscopy (SEM). Zn-1Mg scaffolds were also manufactured and subjected to the same surface treatments. All aforementioned experiments were repeated using Zn-1Mg scaffolds with co-cultured osteoblasts and chondrocytes. **Results**: All samples, irrespective of the surface treatment, showed similar effects compared to the reference surfaces in terms of cell viability, cytotoxicity, and proliferation for both chondrocytes and osteoblasts. SEM analysis revealed comparable cellular morphology across all scaffolds, with cells observed attaching and growing on all scaffold surfaces. This indicates that all scaffolds independent of different surface treatments exhibit good biocompatibility. **Conclusions**: The findings indicate that Zn-1Mg alloy samples with different surface treatments exhibit no significant differences in cytocompatibility with chondrocytes and osteoblasts. Zn-1Mg alloy scaffolds, composed of 99% zinc and 1% magnesium, demonstrate biocompatibility, with cells attaching and growing on all scaffold surfaces. These results suggest that Zn-1Mg alloy scaffolds manufactured additively by PBF-LB/M hold promise for use in resorbable bone graft applications.

## 1. Introduction

Recent advances in 3D printing technologies have significantly expanded their clinical applications in orthopedics, from anatomical modeling to patient-specific implants and customized bone grafts, highlighting the translational potential of additive manufacturing in personalized bone repair and regeneration [1,2]. Zinc-1%-magnesium (Zn-1Mg) alloys have emerged as promising candidates in the field of biomaterials due to their desirable properties, including biocompatibility, mechanical strength, and corrosion resistance [3,4,5]. These alloys hold significant potential for various biomedical applications, particularly orthopedic implants, as they can mimic the mechanical properties of natural bone tissue [6]. However, despite their promising characteristics, the surface properties of such biomaterials are crucial in determining interactions with biological systems [7].

Surface modifications are known to significantly influence cellular responses to biomaterials, impacting key aspects such as adhesion, proliferation, and differentiation. Processing the surface using different methods is almost indispensable for additive manufactured parts, for instance, to remove adhering powder particles. Understanding the effects of different surface treatments on Zn-1Mg alloy scaffolds is essential for optimizing biocompatibility and enhancing performance in bone tissue engineering. While the bulk properties of Zn-1Mg alloys make them attractive for bone implants, surface modifications provide opportunities to tailor interactions with the surrounding tissues and cells [8,9,10].

Various surface treatment techniques, including chemical etching, physical vapor deposition, and plasma treatment, can modify scaffold surface topography, chemistry, and wettability, consequently affecting cellular behavior [11,12,13]. The ability of these modified surfaces to support cell adhesion, proliferation, and differentiation is critical for successful bone regeneration therapies [14,15]. Despite the increasing interest in Zn-1Mg alloys for orthopedic applications, there is a need for comprehensive studies that evaluate the effects of surface treatments and material composition on cellular behavior [16,17].

Chondrocytes and osteoblasts are fundamental cell types involved in cartilage and bone formation, respectively [18]. Investigating the response of these cells to Zn-1Mg alloy scaffolds with different surface treatments provides valuable insights into the material’s biocompatibility and potential for bone tissue engineering [19]. Chondrocytes play a crucial role in maintaining cartilage integrity and function [20,21], while osteoblasts are responsible for bone formation and remodeling [22,23]. Understanding how these cells interact with Zn-1Mg alloy scaffolds can guide the development of implantable devices that promote both cartilage and bone regeneration.

In light of these considerations, this study aims to address current knowledge gaps by investigating the effects of various surface treatments on Zn-1Mg alloy scaffolds. By elucidating the impact of surface modifications on cellular behavior, we seek to advance biomaterials for orthopedic applications, ultimately improving patient outcomes in bone tissue repair and regeneration. It was assumed that the type of Zn-1Mg surface treatment affects the adhesion and proliferation of osteoblasts and chondrocytes to varying degrees.

## 2. Materials and Methods

### 2.1. Preparation of Zn-1Mg Alloy Coins and Zn-1Mg Alloy Scaffolds

**Figure 1:**
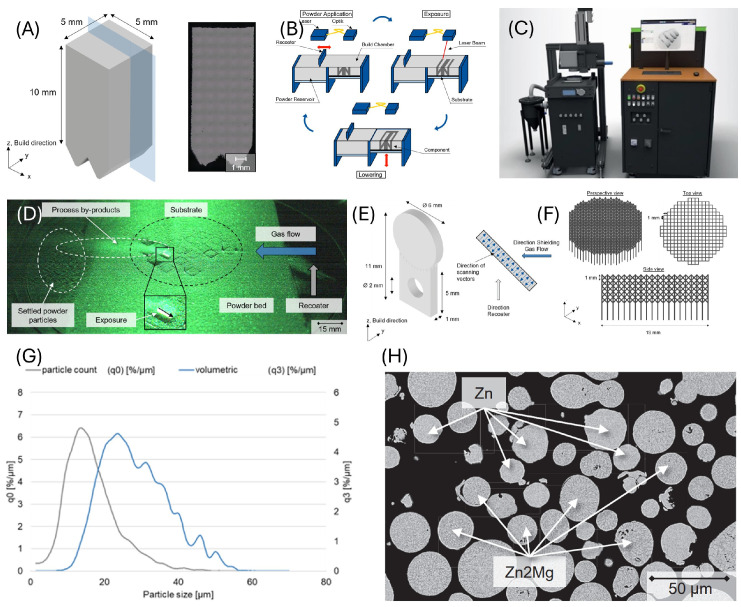
(**A**) The particle size distribution (PSD) both in terms of number (q0) and volume (q3) is shown. Geometrical representation of standard test cube to assess process parameters; metallographic cross-section of manufactured cube from Zn1Mg with a relative density of 99.37 ± 0.07%. (**B**) Schematic working principle of the PBF-LB/M process. (**C**) Aconity Mini machine setup used to manufacture specimens in this work from Zn1Mg. (**D**) The by-products generated during the process, such as metal condensate, resulting from the vaporization of alloying elements during PBF-LB/M. (**E**) Coin specimen geometry. The cytotoxicity of the alloy coins (Ø 6 mm × 1 mm) are additively manufactured. (**F**) Scaffold specimen geometry. A scaffold specimen (Ø 19 mm × 5 mm) was additively manufactured by stacking F2CCZ (Face-Centered Cubic with Z-reinforcement) unit cells (1 × 1 × 1 mm, strut thickness 200 µm) after base removal and post-processing of flat surfaces. (**G**) Particle size distribution measurement of the Zn-1Mg powder used in this work in terms of number (q0) and volume (q3); ordinate: percentage of particles in the corresponding particle size interval. (**H**) SEM images of polished powder grains of commonly used Zn-Mg alloy.

The test specimens discussed in this paper are made of pre-alloyed Zn-1Mg powder from the manufacturer Nanoval GmbH und Co KG (Berlin, Germany) with a particle size distribution (PSD) of +15 µm/−45 µm. The validation of the material specifications is carried out with a particle analyzer of the Camsizer X2 model from the company Retsch (Haan, Germany). The PSD in terms of number (q0) and volume (q3) is shown in Figure 1A.

Additive manufacturing processes are characterized by automated and layer-wise recurring procedures without the use of product-specific tools [24]. The method employed, according to DIN EN ISO/ASTM 52900:2022-03 [25], is referred to as the powder bed fusion of metals using a laser beam (PBF-LB/M), where metal powder is additively processed using laser technology [26]. The PBF-LB/M process is divided into three essential process steps. A schematic representation of the individual steps and terminology is illustrated in Figure 1B. A removable substrate plate made of a specific substrate material is located on a build platform. A layer of powder is applied to the substrate plate at a defined height using a recoater. The required powder is stored in a separate powder reservoir. The laser beam selectively melts areas of the powder bed in the working plane, thereby metallurgically bonding the powder with the substrate plate or later with the most recently melted layers. During exposure, an exposure strategy defined by prior data preparation is executed through the optical system and laser beam [27]. After exposure, the build platform is lowered by a defined layer thickness, followed by another application of powder. Through multiple repetitions of the process steps—powder application, exposure, and lowering—the three-dimensional component is built up layer by layer [28,29].

The test specimens for the experiments in this study were manufactured additively by PBF-LB/M on an AconityMINI system (Aconity 3D GmbH, Herzogenrath, Germany). An overview of the components of this system is shown in Figure 1C. Component implementation and process control are carried out using integrated SLM-Machine software (V3.91 05.02.18).

The test specimens in this study are produced on a building platform with a diameter of 50 mm. The substrate material consists of pure zinc. The powder of the individual layers is applied using a coater with a rubber lip, and excess powder is collected in a removable overflow container at the end of the coating cycle. During the entire build process, the system is under a protective gas atmosphere of argon. Due to the high oxygen affinity of the alloy, the process starts at a residual oxygen content of <200 ppm-O2. This measured value is monitored using a ZR5 oxygen sensor (Zirox, Greifswald, Germany). The system used employs a ytterbium fiber laser, model YLR-400 (IPG Pho-tonics, Burbach, Germany), with a wavelength of 1070 nm. The laser has a maximum output power of 400 W. The laser beam is guided into the optical system via an optical fiber and aligned in parallel using a collimator lens. A hurrySCAN20 galvanic scanner (Scanlab AG, Puchheim, Germany) enables exposure of the entire working plane and has a maximum scanning speed of 4000 mm/s for exposure vectors. An F-Theta lens generates a beam diameter of around 75 µm in the focal plane. The PBF system is equipped with an integrated recirculation and filtration system, allowing by-products generated during the process to be removed via a recirculation flow that runs above the process plane with protective gas, thereby reducing the formation of defects. The generation of smoke and spatter has already been demonstrated for zinc [30], and a protective gas flow has been implemented to minimize the interaction between laser radiation and process by-products [31]. Figure 1D illustrates the by-products generated during the process, such as metal condensate, resulting from the vaporization of alloying elements during PBF-LB/M.

For the experiments in this study, different geometries were prepared. All specimens were manufactured using the same processing parameters as described in [30], which are as follows: volume energy density = 133 J/mm^3^, laser power = 40 W, scanning speed = 200 mm/s, hatch distance = 50 μm, and layer thickness = 30 μm. To investigate the influence of different surface modifications and the cytotoxicity of the material, coins (Ø 6 mm × 1 mm) were additively manufactured (Figure 1E). The attachment of this geometry to the substrate plate was achieved via a 5 mm high base, which supports the lower areas of the coin up to a 45° overhang. The flat areas on the coin faces provided sufficient range for the surface roughness analysis. For cytotoxicity testing, the base was removed so that only the coin remained. After identifying a post-processing route for flat surfaces, a scaffold geometry with a f_2_cc_z_ structure and unit cell size of 1 × 1 × 1 mm was designed. The strut thickness was 200 µm. After arranging the unit cells alongside and on top of each other, a final specimen size of Ø 19 mm × 5 mm was created (Figure 1F). To separate the scaffold from the substrate plate after printing, 3 mm long vertical struts were added at each connection point. Particle size distribution measurement of the Zn-1Mg powder used in this study was performed in terms of number (q0) and volume (q3); ordinate: percentage of particles in the corresponding particle size interval, as shown in Figure 1G. The test parameters used were based on the literature data and were supplemented by additional parameters, as shown in Figure 1H.

### 2.2. Surface Treatments

The factors of surface roughness and possible residues of the printing process and respective modification process can influence behavior during degradation and cell vitality. In the following, the implementation of surface modification using various processes is described. Before any surface modification and subsequent testing, loose powder was removed from all test specimens by an ultrasonic bath in ethanol for 10 min. The surface was then modified using various processes—first the coin geometry and then the scaffold geometry. The structure and execution of these tests are described below. For all tests, an untreated reference sample (reference) was examined in the same amount as the post-processed samples for comparison. The reference was therefore the initial state after the printing process, at room temperature (RT), without any surface modification except for ultrasonic cleaning or contact with cells.

#### 2.2.1. Electrochemical Polishing

The tests in this work were carried out using the electropolishing device LectroPol-5 (Struers GmbH, Willich, Germany). The electrolyte (orthophosphoric acid) was filled into the unit at room temperature (RT) and pre-pumped. A perforated orifice with a diameter of 5 mm was placed at the outlet of the electrolyte. The flow rate of the electrolyte must be adjusted to the orifice plate to cover the sample with sufficient electrolytes during the process. In this case, the flow rate was set to a value of 11. The process voltage was set to 15 V on the device. The test specimens were fixed in place using a crocodile clip. The process was carried out for a duration of 80 s for each side of the coin and 80 s in total for the scaffold geometry. After completion of the electropolishing process, the sample was washed in ethanol and dried in air to stop corrosion reactions.

#### 2.2.2. Coin Particle Blasting

Particle blasting with glass beads has proven to be an efficient method for removing sintering from additively manufactured components due to its pronounced abrasive effect [Li21a]. The tests in this thesis were carried out in a blasting cabinet of type SMG50/1 (MHG Strahlanlagen, Düsseldorf, Germany) using glass beads with particle sizes in the range of 100 µm to 200 µm. This system was equipped with pressure regulation. The components were processed at a distance of around 5 cm from the blasting nozzle tip under altering blasting angles. A blasting pressure of 2 bar was used to reduce the surface roughness without damaging the delicate structures. For this reason, the process duration was limited to 1 min per side for the coin geometry. The scaffold geometry was blasted from all sides for a total of one minute.

#### 2.2.3. Eigengut Blasting

To prevent possible contamination by foreign particles in the glass beads, the effectiveness of Zn-1Mg powder as a blasting material was tested. The particle sizes of the powder used for this purpose were 15–45 µm. The setup and execution of these tests were identical to those for particle blasting. The only difference was the use of an explosion-proof system of type SMG50/1 (MHG Strahlanlagen, Düsseldorf, Germany) due to the low ignition energy of the Zn-1Mg blasting material. Process parameters of 2 bar pressure and 1 min of blasting were identified to treat the surfaces for this group. These process parameters were transferred to the scaffold geometry structures.

#### 2.2.4. Sterilization

Plasma sterilization with hydrogen peroxide was carried out in a STERRAD™ 100NX plasma sterilizer (ASP, Irvine, CA, USA). The standard program had a cycle time of 47 min, reaching temperatures from 47 °C to 56 °C. This system used a combination of hydrogen peroxide vapor and plasma.

#### 2.2.5. Grouping

To facilitate description and analysis, the samples with different surface treatments were categorized into four groups, namely Group A, Group B, Group C, and Group D. Group A corresponds to the reference group, Group B represents the electropolished group, Group C is designated as the particle-blasted group, and Group D refers to Eigengut group. All groups/samples were sterilized with the abovementioned method. We performed cell experiments on all four groups of Zn-1Mg alloy coins first. However, due to the particle-blasting process, the surface of Group C was rough, prone to uneven degradation, and carried a risk of particle detachment. Therefore, it was deemed unsuitable for subsequent animal studies, and Group C was excluded from consideration in the evaluation of cell growth on Zn-1Mg alloy scaffolds.

#### 2.2.6. Surface Analysis

For the qualitative analysis of the surface and roughness measurements, the specimens were placed directly on the microscope slide after the respective surface modification. Overview images and 3D representations of the surface morphology were taken using a VHX-6000 reflected light microscope (LM) from Keyence (Osaka, Japan). Due to the different nature of the surfaces, different exposures and magnifications were selected to emphasize the respective features. To quantify the surface modifications, the line roughness of the specimens was measured. This was also carried out on the LM and can therefore be classified as an optical roughness measurement. An automated program was used to record and compile images in different focal planes. A height profile was created from this. In order to generate comparable values for the arithmetic average roughness R_a_ and the average roughness depth R_z_, the low-pass filter λ_c_ was set to 0.8 mm. The statistical reliability of the values obtained was ensured with three measurements per test specimen.

Qualitative detailed images of the surface were taken using SEM. The conductivity of the test specimens was ensured using copper tape. The acceleration voltage for these measurements was 10 kV at magnifications ranging from 100× to 50,000×.

### 2.3. Scanning Electron Microscopy (SEM) Analysis

In order to further observe the microscopic surface morphology of Zn-1Mg coins and Zn-1Mg scaffolds, as well as the attachment of osteoblasts and chondrocytes on the Zn-1Mg scaffolds, we used scanning electron microscopy (SEM). For SEM, cells were fixed with 3% (vol/vol) glutaraldehyde (Agar Scientific, Wetzlar, Germany) in 0.1 M Soerensen’s phosphate buffer, washed in phosphate buffer for 15 min, and dehydrated by incubating consecutively in an ascending ethanol series (30%, 50%, 70%, 90%, and 100%) for 10 min each and the last step thrice. The samples were air dried and coated (Sputter Coater EM SCD500, Leica, Wetzlar, Germany) with a 5 nm carbon layer. Samples were analyzed in backscatter mode using a scanning electron microscope (Quattro S, Thermo Fisher) at 10 kV acceleration voltage in a high-vacuum environment. The mineral distribution within the aggregates was analyzed using SEM in combination with energy-dispersive X-ray (SEM-EDX) analysis [32].

### 2.4. Evaluation of Cell Growth DAPI Staining

To better simulate the in vivo osteoinductive environment of Zn–Mg alloy coins and scaffolds, they were, respectively, incorporated into wells containing osteoblasts and chondrocytes. To prepare the growth medium for osteoblasts, DMEM (Pan Biotech, Aidenbach, Germany, catalog number: P04-05550) supplemented with 10% heat-inactivated fetal bovine serum (FBS) (Gibco, Grand Island, NY, USA, catalog number: 26140079) and 1% penicillin–streptomycin (Gibco, catalog number: 2257214) was used. For chondrocytes, the growth medium was prepared with DMEM/F-12(Gibco, Grand Island, NY, USA, catalog number: 11320033) supplemented with 10% heat-inactivated fetal bovine serum (FBS) (Gibco, Grand Island, NY, USA, catalog number: 26140079) and 1% penicillin–streptomycin (Gibco, Grand Island, NY, USA, catalog number: 2257214). Next, the osteoblasts and chondrocytes were separately cultured in tissue culture dishes (TPP, Trasadingen, Switzerland, catalog number: 90076) containing growth medium and maintained at 37 °C in a humidified 5% CO_2_ atmosphere. Isolated cells were passaged until passage 3~5 for subsequent experiments.

Osteoblasts and chondrocytes were separately seeded onto 6-well plates at a density of 2 × 10^4^ cells per well with Zn–Mg alloy coins inside. The cells were cultured in growth medium and maintained in a humidified incubator with 5% CO_2_ at 37 °C for 3 days, 7 days, and 14 days. Following the respective culture periods, cells were washed 1–3 times with phosphate-buffered saline (PBS) to remove residual growth medium. DAPI staining dye was used at 10 μg/mL, the final concentration. The cells were then incubated for 3 min, protected from light, to allow for nuclear staining. After the incubation period, the DAPI stain solution was carefully removed, and the cells were washed 2–3 times with PBS to remove excess staining. Following the final PBS wash, the cells were imaged using fluorescence microscopy (Leica DMI 4000B, Wetzlar, Germany) to visualize the stained nuclei.

For SEM observation of the Zn–Mg alloy coins, the coins were placed in a 24-well plate, and the cell–hydrogel suspension (at a density of 2 × 10^4^ cells/mL by mixing 4 × 10^4^ cells with 1 mL of culture medium and 1 mL of hydrogel) was added to completely cover the coin surface. After 7 days of culture, the samples were processed for observation. For the Zn–Mg alloy scaffolds, the osteoblasts and chondrocytes were prepared at a density of 2 × 10^4^ cells/mL. The scaffolds were placed in a 6-well plate, and the cell suspension was added to completely cover the top of the scaffold. The cells were cultured for 7 days, after which they were processed for scanning electron microscopy (SEM) observation.

### 2.5. Evaluation of Cell Morphology Phalloidin/DAPI Staining

For cytoskeleton staining, osteoblasts and chondrocytes were seeded onto 6-well plates at a density of 20,000 cells per well. The cells were cultured in growth medium and maintained in a humidified incubator with 5% CO_2_ at 37 °C. After incubation for 14 days, we assessed cell morphologies with different scaffolds. Osteoblasts and chondrocytes were separately subjected to co-staining with Rhodamine Phalloidin (ThermoFisher, Grand Island, NY, USA) and DAPI, which aids in visualizing F-actin and cell nuclei. Briefly, osteoblasts and chondrocytes were rinsed with DPBS and fixed in 4% paraformaldehyde, followed by permeabilizing with 0.1% Triton X-100 and blocking with 1% BSA solution [26,27]. After that, osteoblasts and chondrocytes were co-stained with the indicated fluorescent regents and visualized via a CLSM under Ex/Em (540/565 nm) wavelength.

### 2.6. Flow Cytometry

Osteoblasts and chondrocytes were seeded onto 6-well plates at a density of 20,000 cells per well and cultured for 3 days. Trypsin was used to digest the cells, then centrifugation was used to remove the supernatant, and the cell pellet was collected. Zombie Violet fixable viability kit (Biolegend, London, UK, catalog number: 423113) was prepared at a 1:2000 dilution in PBS, resulting in a final volume of 1 mL. The prepared solution was added to the cell suspension, covering the cells entirely [33,34,35,36]. The cell suspension was gently mixed and incubated at 37 °C in the dark for 30 min to allow for cell staining. Following the incubation, stained cells were washed three times with PBS to remove excess dye. The fluorescence of 10,000 cells was analyzed using a flow cytometer. FlowJo v10.7 software (BD Life Sciences, Franklin Lakes, NJ, USA) was utilized to calculate the ratio of live cells based on the fluorescence data obtained from the flow cytometer analysis [37].

### 2.7. Statistical Analysis

Experimental data are shown as the mean ± standard deviation (SD). Statistical analysis was carried out using SPSS 22.0 software (Armonk, NY, USA) and ORIGIN 9.0 software (Northampton, MA, USA). Student’s *t*-test and two-way ANOVA were applied to compare the significance among two groups or multiple groups. *p* < 0.05 was considered as a minimal level of significance, *p* < 0.01 was regarded as a medium level of significance, and *p* < 0.001 was indicated as a high level of significance.

## 3. Results

### 3.1. Additive Manufacturing of Specimen

The processing of zinc-magnesium alloys using PBF-LB/M presents significant challenges due to their physicochemical properties. The high vapor pressure at low melting temperatures and the narrow temperature window between melting and evaporation lead to an unusually high number of process by-products, resulting in a limited process window. Based on preliminary investigations [30], specimens with high relative density (>99.5%) can be manufactured. A cross-section of Zn1Mg cubic specimens is shown as an example in Figure 1A. In addition, sintered particles can be observed on the edges and the overhang areas of the sample. Nevertheless, this parameter set was chosen to manufacture all the geometries in this study.

After the manufacturing process, the surface of the reference geometry shows process-related sintering. Figure 1E compares the surface roughness achieved after the modifications with the reference. Electropolishing with 15 V and 80 s on each side can reduce the surface roughness by around 29% to R_a_ 6.55 µm, but not all surface-sintered particles can be removed. While the surface still resembles the reference after electropolishing, the surface morphology changes after modification using the particle-blasting methods. All surface-sintered particles can be removed with both particle materials. Sandblasting results in a surface with the lowest roughness of all the methods tested in this work, with R_a_ 1.08 µm, a reduction of around 88% compared to the reference. With intrinsic particle blasting, the surface roughness can be reduced by around 61% to R_a_ 3.57 µm.

**Figure 2:**
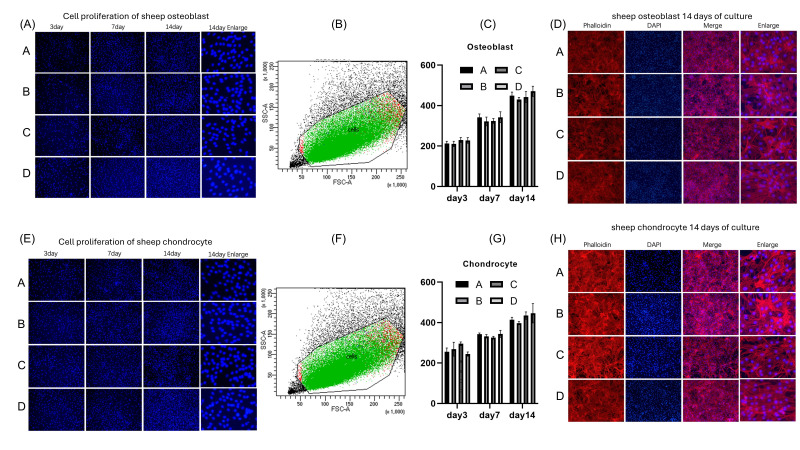
(**A**) Osteoblasts treated with four different materials were stained with DAPI on days 3, 7, and 14. Fluorescence microscopy images revealed that the nuclei of all cells were clearly visible at each time point. Additionally, there was a noticeable increase in the number of nuclei over time. (**B**) Green fluorescence indicated live osteoblasts, red fluorescence indicated dead osteoblasts, while black dots represented non-cellular particles. The figure showed a high survival rate across all four groups. (**C**) No significant statistical difference was found on day 3, day 7, and day 14. Osteoblast Group A (334.3 ± 118.2), Group B (320.7 ± 109.8), Group C (332.1 ± 106.5), Group D (346.8 ± 121.6); *p* > 0.05. (**D**) Cell morphological observations of osteoblast growth with various scaffolds after 14 days of cultivation via CLSM. Osteoblasts were fluorescently labeled by cytoskeletal staining with Rhodamine Phalloidin and DAPI, which indicate F-actin (red) and cell nuclei (blue). Scale bar: 50 or 20 µm. (**E**) Chondrocytes treated with four different surface morphologies were stained with DAPI on days 3, 7, and 14. Fluorescence microscopy images revealed that the nuclei of all cells were clearly visible at each time point. Additionally, there was a noticeable increase in the number of nuclei over time. (**F**) Green fluorescence indicated live chondrocytes, red fluorescence indicated dead chondrocytes, while black dots represented non-cellular particles. The figure showed a high survival rate across all four groups. (**G**) No significant statistical difference was found on day 3, day 7, and day 14. Chondrocyte Group A (337.4 ± 79.63), Group B (332.7 ± 64.17), Group C (351.9 ± 73.27), Group D (345.0 ± 100.7); *p* > 0.05. (**H**) Cell morphological observations of chondrocyte growth with various scaffolds after 14 days of cultivation via CLSM. Osteoblasts were fluorescently labeled by cytoskeletal staining with Rhodamine Phalloidin and DAPI, which indicate F-actin (red) and cell nuclei (blue). Scale bar: 50 or 20 µm.

**Figure 3:**
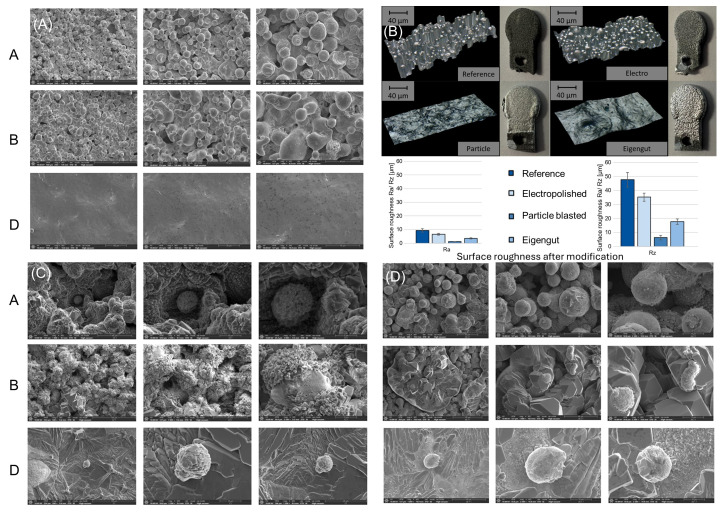
(**A**) Scanning electron microscopy (SEM) images of three different material surface conditions without cells. Group A (reference) indicates an untreated, highly textured, and porous surface. Group B (electropolished) demonstrates a highly textured surface with visible round features and a porous structure. The surfaces have undergone an electropolishing process, resulting in the removal of surface material and the formation of a uniform but rough texture with many depressions and elevations. Group D (Eigengut) exhibits a highly uniform and smooth surface with minimal microstructural irregularities and no observable protrusions or sharp edges. The surface topography is flat and continuous, with low roughness, forming a striking contrast to the rough surfaces of Groups A and B. (**B**) Representative images of the four materials with different surface treatments, along with the presentation of their surface roughness characteristics. (**C**) Representative SEM images of chondrocytes with various scaffolds after 14 days of cultivation. Scale bar: 100 µm. (**D**) Representative SEM images of osteoblasts with various scaffolds after 14 days of cultivation. Scale bar: 100 µm. Group A corresponds to the reference group, Group B represents the electropolished group, and Group D refers to the intrinsic material blasted group. All groups/samples were sterilized with the abovementioned method.

Figure 2 shows the co-culture of Zn-1Mg coins with osteoblasts and chondrocytes, and Figure 3 shows scanning electron microscopy (SEM) images, which allow for a detailed examination of the surface morphology. The analysis shows clear differences in different states. While a large number of surface-sintered powder particles can be seen in the reference geometry and the electropolished one, these can be completely removed by particle blasting using both glass beads and the powder itself. A significant difference between the two methods lies in the detail of the surface properties, particularly the surface roughness. The sandblasted surface is actually very smooth, with minimal deformations or edges, resulting in a polished appearance.

### 3.2. Zn-1Mg Alloy Coins

#### 3.2.1. Zn-1Mg Coin Surface Observation

In order to better observe the morphology of the Zn-1Mg coin, we conducted observations (Figure 3A) of scanning electron microscopy (SEM) images of three different material surface conditions without cells. Group A indicates a highly textured and porous surface, similar to electropolished surfaces but with more irregularity and larger, more prominent spherical features. Group B demonstrates a highly textured surface with visible rounded features and a porous structure. The surfaces have undergone an electropolishing process, resulting in the removal of surface material and the formation of a uniform but rough texture with many depressions and elevations. Group D indicates that the surfaces appear homogenous with minimal texture.

#### 3.2.2. Co-Culture of Zn-1Mg Coins with Osteoblasts and Chondrocytes

Figure 2A,E shows that the number of cells increased as time went by from 3 days to 7 days and 14 days. The cell nucleus had regular morphology and the cells on the surface grew healthily. We then used ImageJ (version: 1.53v) software to calculate the difference in cell number between the groups. As shown in Figure 2C,G, there was no statistical difference in the growth of osteoblasts and chondrocytes among the groups. We then used Zombie Violet to mark dead cells and performed flow cytometry analysis. Figure 2B,F shows that green fluorescence indicated live osteoblasts, red fluorescence indicated dead osteoblasts, while black dots represented non-cellular particles. The figure shows a high survival rate across all four groups.

To further observe the growth status of cells, cell morphological observations of chondrocyte growth with various scaffolds after 14 days of cultivation via CLSM were performed. Osteoblasts and chondrocytes were fluorescently labeled by cytoskeletal staining with Rhodamine Phalloidin and DAPI, which indicate F-actin (red) and cell nuclei (blue) (Figure 2D,H). Scale bar: 50 or 20 µm. The results showed that in each group, osteoblasts and chondrocytes grew well, with clear intracellular F-actin staining and regular cell nuclear morphology.

#### 3.2.3. SEM Analysis of Co-Culture of Coins with Osteoblasts and Chondrocytes

To better observe the interaction between Zn-1Mg coins and co-cultured chondrocytes and osteoblasts, we performed direct SEM imaging of the cells on the Zn-1Mg coin surfaces. Figure 3C indicates the result of Zn-1Mg coins co-cultured with chondrocytes, while Figure 3D shows Zn-1Mg coins co-cultured with osteoblasts. SEM analysis provides direct evidence of the attachment of both chondrocytes and osteoblasts on Zn-1Mg coins in all surface treatment groups. This indicates that the surface treatment of Zn-1Mg coins does not significantly affect the growth or adhesion of either cell type.

### 3.3. Zn-1Mg Scaffolds

#### 3.3.1. Zn-1Mg Scaffold Surface Observation

**Figure 4:**
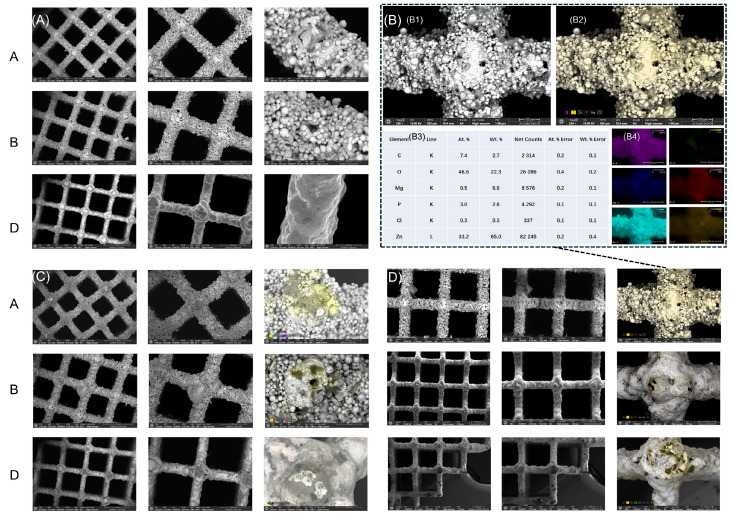
(**A**) SEM images of Zn-1Mg alloy scaffolds after different surface treatments, demonstrating distinct morphological features. Group A: Surface exhibited pronounced roughness with irregular morphology, dense particle coverage, and visible porosity. Group B: Surface roughness was slightly reduced compared to Group A, but numerous granular protrusions remained, indicating a still relatively coarse texture. Group D: Surface appeared markedly smoother than in Groups A and B, with minimal granular structures and nearly smooth morphology. (**B**) (**B1**) The reference scaffold SEM observation. (**B2**) The reference scaffold SEM-EDX staining. (**B3**) SEM-EDX elemental analysis and energy-dispersive X-ray spectroscopy. (**B4**) Analysis of particle elements using EDX spectrometric maps. Yellow area contains carbon, oxygen, magnesium, phosphorus, chlorine, and zinc. SEM/EDX elemental analysis results showed that there was a large amount of organic matter attached to the surface of the scaffold, indicating that a large number of cells were growing. (**C**) Representative SEM images of chondrocytes with various scaffolds after 14 days of cultivation. Scale bar: 100 µm. (**D**) Scanning electron microscopy (SEM) images of the porous metal scaffolds revealed the presence of osteoblasts on the surfaces of each scaffold, providing direct evidence of cell adhesion to the scaffold materials. Additionally, energy-dispersive X-ray spectroscopy (EDX) was used to stain the cells yellow, allowing for clear differentiation between the cells and other inorganic materials. The yellow regions in the images confirmed that the observed material was indeed cellular, demonstrating that all Zn-1Mg scaffolds exhibit good biocompatibility.

To further analyze the scaffold surface morphology, we performed scanning electron microscopy (SEM). As shown in Figure 4A, the SEM images of Zn-1Mg alloy scaffolds reveal distinct characteristics for each surface treatment. Group A: The surface exhibited pronounced roughness with irregular morphology, dense particle coverage, and visible porosity. Group B: Surface roughness was slightly reduced compared to Group A, but numerous granular protrusions remained, indicating a still relatively coarse texture. Group D: The surface appeared markedly smoother than in Groups A and B, with minimal granular structures and nearly smooth morphology.

#### 3.3.2. Co-Culture of Zn-1Mg Scaffolds with Osteoblasts and Chondrocytes

To further observe the performance of the osteoblast and chondrocyte growth on the scaffolds, SEM was conducted. Figure 4C shows the representative SEM images of chondrocytes with various scaffolds after 14 days of cultivation. Figure 4D shows that the SEM images of the porous metal scaffolds revealed the presence of osteoblasts on the surfaces of each scaffold. This provided direct evidence of cell adhesion to the scaffold materials. Additionally, an analysis of particle elements using EDX spectrometric maps is shown. The yellow area contains carbon, oxygen, magnesium, phosphorus, chlorine, and zinc. SEM/EDX elemental analysis results of Group A with osteoblast cells showed that there was a large amount of organic matter attached to the surface of the scaffold, indicating validation of the successful attachment of osteoblasts to the Zn-1Mg scaffolds.

## 4. Discussion

In this study, we aimed to evaluate the potential medical application of Zn-1Mg alloy scaffolds by assessing their effects on chondrocytes and osteoblasts in terms of cytotoxicity, biocompatibility, and cell growth. Our experimental approaches involved designing Zn-1%-Mg alloy coins, initially analyzing their surface morphology using electron microscopy and subsequently co-culturing them with chondrocytes and osteoblasts. SEM images of Zn-1Mg alloy scaffolds after different surface treatments demonstrated distinct morphological features.

The initial set of experiments focused on assessing cell viability, toxicity, and proliferation over 3, 7, and 14 days. Flow cytometry using live/dead staining indicated high cell survival rates across all scaffold treatments, suggesting that the scaffolds did not adversely affect cell viability. DAPI staining at different time points demonstrated healthy cell proliferation, also confirming that the scaffolds did not inhibit cell growth. Fluorescence microscopy images were obtained for cells treated with four different materials and stained with Phalloidin/DAPI. The images demonstrated that the nuclei in all treatment groups were clearly visible, and actin filaments were distinctly stained, indicating successful staining. Moreover, the overall morphology of the cells appeared healthy across all groups. These observations suggest that none of the tested materials exert a negative impact on cellular growth. There was no evidence of cytotoxicity, and the materials did not interfere with cell proliferation, which matches the previous studies [38,39]. No significant statistical differences were observed among the groups with different surface treatments, indicating that there was no statistical difference in the effects of Zn-1Mg coins with different surface treatments on cells. These findings collectively indicate that the four materials are biocompatible and suitable for use in further cellular applications.

To further investigate the biocompatibility of Zn-1Mg alloys, we redesigned the scaffolds into larger porous structures so that cells can better adhere to the scaffold surface. The SEM images of Zn-1Mg alloy scaffolds show distinct features for each surface treatment.

DAPI staining again showed healthy cell proliferation at various time points, and Phalloidin/DAPI staining after 14 days indicated robust cytoskeletal development. These findings were consistent with the results obtained from the initial coin experiments, reinforcing the notion that the Zn-1Mg scaffolds did not exert any negative impact on cell growth and viability.

SEM images of the porous Zn-1Mg scaffolds revealed the presence of osteoblasts or chondrocytes on the surfaces of each scaffold. This provides direct evidence of cell adhesion to the scaffold materials. In Figure 3, we observed tiny, regular spherical structures with diameters ranging from approximately 20 nm to 100 nm. After repeated experiments and extensive testing, we confirmed that these small structures originate from hydrogel degradation products rather than contamination. Control samples were also analyzed, ensuring that these observations were not due to external contaminants, thus eliminating potential reviewer concerns regarding contamination artifacts. Additionally, energy-dispersive X-ray spectroscopy (EDX) was used to stain the cells yellow, allowing for clear differentiation between the cells and other inorganic materials. The yellow regions in the images confirm that the observed material is indeed cellular, thereby validating the successful attachment of osteoblasts to the Zn-1Mg scaffolds.

All scaffolds supported cell attachment and growth, as evidenced by the presence of osteoblasts and chondrocytes on the material surfaces. The proliferation assays consistently showed no significant adverse effects on the cells, highlighting the biocompatibility of the Zn-1Mg alloy scaffolds. Zinc and magnesium ions released during alloy degradation play essential roles in modulating cellular behavior and tissue regeneration. Zn^2+^ is a vital trace element involved in numerous enzymatic reactions, transcriptional regulation, and cell cycle progression. Moderate concentrations of Zn^2+^ have been shown to enhance osteoblast proliferation and differentiation by activating MAPK/ERK and Wnt/β-catenin signaling pathways, thereby promoting matrix mineralization and bone formation [40,41]. However, excessive Zn^2+^ release can lead to cytotoxicity due to local acidification and oxidative stress [42]. Similarly, Mg^2+^ ions are critical cofactors for ATP-dependent enzymes and nucleic acid synthesis, and they stabilize cell membranes while supporting mitochondrial activity. Controlled Mg^2+^ release has been reported to stimulate integrin-mediated adhesion and PI3K/Akt signaling, enhancing osteogenic cell proliferation and spreading [43,44]. Therefore, the balanced degradation of Zn–Mg alloys provides a synergistic ionic microenvironment that supports cellular metabolism, proliferation, and early osteogenic responses, which is crucial for optimal bone regeneration.

This study was limited to in vitro experiments and did not include animal experiments; therefore, no data on morphological evolution of the Zn–Mg alloy in vivo are available. Previous in vivo investigations have shown that Zn–Mg-based implants undergo progressive morphological changes after implantation, with surface roughness initially increasing due to localized corrosion and pitting, followed by the formation of a more compact and bone-integrated corrosion layer over time [45,46]. Similarly, studies have reported that surface topography strongly influences corrosion kinetics and ion exchange behavior in physiological environments, where higher surface roughness accelerates ion release and promotes calcium–phosphate deposition [47]. Future work should include in vivo evaluation to better understand how degradation-induced surface morphology affects ion release, bone integration, and overall biological performance.

In this study, osteoblasts and chondrocytes were employed to evaluate the cytocompatibility of Zn–Mg alloy coins and scaffolds, as they are directly relevant to bone and cartilage regeneration. Nevertheless, macrophages, fibroblasts, and endothelial cells play critical roles in the osteogenic microenvironment, including inflammatory responses and angiogenesis. Future studies should incorporate these cell types to provide a more comprehensive assessment of material–tissue interactions and regenerative potential.

In conclusion, our comprehensive analysis of Zn-1Mg alloy scaffolds demonstrates their suitability for biomedical use, particularly in applications requiring biocompatible materials that support cell proliferation and function. Future research could explore the long-term effects of these scaffolds in vivo, as well as their potential for integration into more complex tissue engineering frameworks.

## Data Availability

The original contributions presented in the study are included in the article. Further inquiries should be directed to the corresponding authors.
